# Genome‐wide association mapping of the architecture of susceptibility to the root‐knot nematode *Meloidogyne incognita* in *Arabidopsis thaliana*

**DOI:** 10.1111/nph.15034

**Published:** 2018-02-22

**Authors:** Sonja Warmerdam, Mark G. Sterken, Casper van Schaik, Marian E. P. Oortwijn, Octavina C. A. Sukarta, Jose L. Lozano‐Torres, Marcel Dicke, Johannes Helder, Jan E. Kammenga, Aska Goverse, Jaap Bakker, Geert Smant

**Affiliations:** ^1^ Laboratory of Nematology Wageningen University Droevendaalsesteeg 1 6708 PB Wageningen the Netherlands; ^2^ Laboratory of Plant Breeding Wageningen University Droevendaalsesteeg 1 6708 PB Wageningen the Netherlands; ^3^ Laboratory of Entomology Wageningen University Droevendaalsesteeg 1 6708 PB Wageningen the Netherlands

**Keywords:** allelic variation, *Arabidopsis thaliana*, brassinosteroid signalling, F‐box protein, genome‐wide association, *Meloidogyne incognita*, susceptibility

## Abstract

- Susceptibility to the root‐knot nematode *Meloidogyne incognita* in plants is thought to be a complex trait based on multiple genes involved in cell differentiation, growth and defence. Previous genetic analyses of susceptibility to *M. incognita* have mainly focused on segregating dominant resistance genes in crops. It is not known if plants harbour significant genetic variation in susceptibility to *M. incognita* independent of dominant resistance.
- To study the genetic architecture of susceptibility to *M. incognita*, we analysed nematode reproduction on a highly diverse set of 340 natural inbred lines of *Arabidopsis thaliana* with genome‐wide association mapping. We observed a surprisingly large variation in nematode reproduction among these lines.
- Genome‐wide association mapping revealed four quantitative trait loci (QTLs) located on chromosomes 1 and 5 of *A. thaliana* significantly associated with reproductive success of *M. incognita*, none of which harbours typical resistance gene homologues. Mutant analysis of three genes located in two QTLs showed that the transcription factor BRASSINAZOLE RESISTANT1 and an F‐box family protein may function as (co‐)regulators of susceptibility to *M. incognita* in Arabidopsis.
- Our data suggest that breeding for loss‐of‐susceptibility, based on allelic variants critically involved in nematode feeding, could be used to make crops more resilient to root‐knot nematodes.

## Introduction

Polyphagous root‐knot nematodes significantly undermine agricultural productivity in major food crops worldwide (Jones *et al*., 2013). In a recent study on biotic risk factors of global food security, the tropical root‐knot nematode *Meloidogyne incognita* was ranked as the most invasive plant disease‐causing agent (Bebber *et al*., 2014). For decades, root‐knot nematode infestations have been controlled by applications of chemical pesticides. However, most pesticides against root‐knot nematodes face regulatory bans owing to their high human and environmental toxicity. The phasing‐out of chemical pesticides to root‐knot nematodes has significantly increased the global demand for nematode‐resistant crops. However, for only a few crops, such as tomato, prune, carrot and pepper, highly specific dominant resistance genes against root‐knot nematodes are available (Williamson & Kumar, 2006; Davies & Elling, 2015).

Two natural phenomena currently threaten the use of dominant resistances to root‐knot nematodes, the first of which is genetic selection for resistance‐breaking nematode populations. For instance, most of the commercial cultivars of tomato (*Solanum lycopersicum*) carry introgressions of the dominant *Mi‐1.2* gene from the wild tomato species *Solanum peruvianum*, which in many areas is no longer able to confer high levels of resistance to several tropical root‐knot nematode species (e.g. *M. incognita*,* Meloidogyne javanica* and *Meloidogyne arenaria*; Castagnone‐Sereno *et al*., 2013). Findings of virulent field populations of *M. incognita* in tomato with the *Mi‐1.2* gene are not a particularly recent development (Kaloshian *et al*., 1996; Semblat *et al*., 2000). However, their widespread dispersal across major tomato‐producing regions has lately turned them into a major concern for growers. Second, many known dominant resistances to tropical root‐knot nematodes are temperature sensitive and rising soil temperatures by global warming may render them ineffective (Jacquet *et al*., 2005).

Root‐knot nematodes are obligate biotrophs that feed for weeks on living cells within the vascular cylinder of the root of a host plant. Soil‐borne second‐stage juveniles (J2s) of *M. incognita* invade the roots at the transition zone close to the root tip. The J2s then migrate intercellularly through the root cortex towards the root meristem, where they enter the vascular cylinder from below. Inside the vascular cylinder the J2s establish a permanent feeding structure consisting of several giant nurse cells (Caillaud *et al*., 2008). For the initiation of these giant cells, the J2s redirect the differentiation and growth of vascular cells into large transfer cell‐like units. The exact molecular mechanisms underlying the cellular transformation of vascular parenchyma into giant cells are not well understood. However, it is clear that giant cell formation involves alterations in a wide range of fundamental molecular and cellular processes, including epigenetic control of gene expression, cell cycle regulation, plant cell wall modifications and cytoskeletal rearrangements (Kyndt *et al*., 2013). Prolonged feeding on giant cells enables the J2s to moult three times into the adult female stage. After a couple of weeks, adult female root‐knot nematodes produce offspring as an aggregate of eggs held together by a gelatinous matrix (Kyndt *et al*., 2013).

Giant cells are a polygenic trait of nematode‐infected plants, involving hundreds of different plant genes. Studies on giant cell‐enriched root tissue from *Arabidopsis thaliana* infected with *M. incognita* revealed > 3000 differentially regulated genes in a comparison with uninfected root tissue (Jammes *et al*., 2005). Similarly, *c*. 1000 genes appeared to be differentially regulated in giant cell‐enriched tissue of *M. incognita* at 21 d post‐inoculation in Arabidopsis compared to uninfected tissue (Fuller *et al*., 2007). A similar number of differentially expressed genes were identified in a comparison of microdissected giant cells and neighbouring vascular cells in Arabidopsis at 3 d post‐inoculation with the tropical root‐knot nematode *M. javanica* (Barcala *et al*., 2010). Although not all genes regulated in association with giant cell formation will be causally linked to this process, allelic variation in specific subsets of these genes may quantitatively affect the susceptibility of a host plant to root‐knot nematodes.

Quantitative traits can be mapped onto specific genome loci by exploiting linkage disequilibrium (LD) between allelic variants (i.e. single nucleotide polymorphisms (SNPs)) and a particular trait in a set of individuals. Genome‐wide association (GWA) mapping expands on this principle by studying associations between a large number of SNPs across a genome and complex traits within a sample of genetically diverse individuals from a natural population (Zhu *et al*., 2008). At present, the richest resources for GWA mapping between SNPs and complex traits in plants focus on large collections of natural inbred lines of Arabidopsis (Atwell *et al*., 2010; Cao *et al*., 2011; Weigel, 2012). Arabidopsis serves as a model organism to study plant responses to all kinds of abiotic and biotic stresses, including infections by root‐knot nematodes (Sijmons *et al*., 1991). Genome‐wide associations between allelic variants and responses to a variety of biotic and abiotic stresses have recently been mapped onto the genome of Arabidopsis (Kloth *et al*., 2012, 2016; Bac‐Molenaar *et al*., 2015; Davila Olivas *et al*., 2017). Moreover, multi‐trait genome‐wide association mapping has been used to reveal cross‐correlations between SNPs and resistances to different biotic and abiotic stresses in Arabidopsis, including parallels in responses to osmotic stress and root‐knot nematodes (Thoen *et al*., 2017).

In theory, plants could be made more resistant to nematode infections by selecting for less conducive allelic variants of genes that critically determine susceptibility (i.e. *S*‐genes; de Almeida Engler *et al*., 2005; van Schie & Takken, 2014). Given the problems with dominant resistance genes in food crops, we asked whether plants harbour significant natural variation in susceptibility to root‐knot nematodes, which is not related to dominant resistance. Here, we present the results of a GWA study of quantitative variation in susceptibility to the root‐knot nematode *M. incognita* in Arabidopsis. Our primary interest was to analyse allelic variation in genes that do not resemble major resistance gene homologs. For this, we chose to work with Arabidopsis, because previous research suggested that dominant resistance to *M. incognita* may be absent in this species (Niebel *et al*., 1994). In fact, it is not so likely that the resistance gene repertoire of Arabidopsis, with its native range in more temperate regions of Europe and Asia (Beck *et al*., 2008), has undergone extensive adaptations to tropical root‐knot nematodes (e.g. *M. incognita*). Natural Arabidopsis inbred lines are also particularly well suited for GWA mapping of disease susceptibility, because they allow for repeatedly phenotyping of genetically identical individuals in notoriously variable *in vitro* bioassays with nematodes. In total, we found eight SNPs in our GWA study to be significantly associated with the reproductive rate of *M. incognita* in 340 Arabidopsis lines. By using the predicted LD decay for the Arabidopsis genome, we aggregated the SNPs into four genomic regions, two of which we examined in more detail in this paper. Our data on the candidate genes in these loci demonstrate that the transcription factor BRASSINAZOLE RESISTANT‐1 and an F‐box family protein in Arabidopsis probably (co‐)regulate susceptibility to *M. incognita*.

## Materials and Methods

### Plant material

For genome‐wide association mapping, we used a population consisting of 340 natural inbred lines selected from a global HapMap collection of *Arabidopsis thaliana* (L.) Heynh. (http://bergelson.uchicago.edu/wp-content/uploads/2015/04/Justins-360-lines.xls). The homozygous T‐DNA insertion mutant lines Salk_052305C (hereafter referred to as *gsp1‐1*) and Salk_050274C (hereafter *frni1‐1*), the ethyl methanesulfonate (EMS)‐induced mutant line *bzr1‐1D*, and the *BZR1:CFP* gene fusion reporter line were obtained from the Nottingham *Arabidopsis* Stock Centre (Alonso *et al*., 2003). The *bzr1‐1D* and *BZR1:CFP* lines were originally described by Wang *et al*. (2002). The *bzr1‐1D*,* BZR1:CFP*,* gsp1‐1* and *frni1‐1* lines were all generated in the background of *A. thaliana* Col‐0.

The homozygosity of T‐DNA inserts was checked by PCR on genomic DNA isolated from leaf material (Holterman *et al*., 2006) of 12 seedlings using primer combinations as indicated in Supporting Information Table S1. The following conditions were used for PCR: 10 min at 94°C, 35 cycles of 30 s at 94°C, 1.5 min at 60°C and 1 min at 72°C, and a final incubation of 10 min at 72°C. The PCR amplification products were analysed by agarose gel electrophoresis.

### Nematode infection assays

Eggs of *M. incognita* were obtained by treating tomato roots infected with *M. incognita* (strain ‘Morelos’ from INRA, Sophia Antipolis, France) with 0.05% (v/v) NaOCl for 3 min. Roots were rinsed with tap water and the eggs were collected on a 25 μm sieve. Next, the eggs were incubated in a solution of 2.4 mM NaN_3_ for 20 min with shaking. Thereafter, the eggs were rinsed with tap water and incubated on a 25 μm sieve in a solution of 1.5 mg ml^−1^ gentamycin and 0.05 mg ml^−1^ nystatin in the dark at room temperature. Hatched juveniles were collected after 4 d and surface sterilized (0.16 mM HgCl_2_, 0.49 mM NaN_3_, 0.002% (v/v) Triton X‐100) for 10 min. After surface sterilization, the juveniles were rinsed three times with sterile tap water and transferred to 0.7% Gelrite solution (Duchefa Biochemie, Haarlem, the Netherland).

To generate cultures of Arabidopsis seedlings *in vitro*, seeds were vapour‐sterilized (in 0.7 M NaOCl and 1% HCl in tap water) for 5 h and transferred to a six‐well cell culture plate containing Murashige and Skoog (MS) medium with vitamins 4.7 g l^−1^ (Duchefa Biochemie), 58.5 mM sucrose and 5 g l^−1^ Gelrite (Duchefa Biochemie). The six‐well plates with seeds were incubated in the dark at 4°C for 3 d. Next, the seeds were allowed to germinate at 21°C under 16 h : 8 h, light : dark conditions. To determine the susceptibility of the 340 natural Arabidopsis inbred lines and the *bzr1‐1D*,* gsp1‐1* and *frni1‐1* mutant lines, 1‐wk‐old seedlings were manually transferred to wells in a new six‐well plate containing MS medium and incubated for another 7 d at 21°C under a 16 h : 8 h, light : dark regime. Each well contained only one seedling. Next, the seedlings were inoculated with 180 infective J2s of *M. incognita* per plant and incubated at 24°C in the dark.

To be able to count the number of infective juveniles in the *bzr1‐1D*,* gsp1‐1* and *frni1‐1* mutant lines at 7 d after inoculation, whole roots were stained with acid fuchsin. To this end, clean roots were first incubated in 16.8 mM NaOCl for 5 min, and thereafter in tap water for 10 min. Next, the roots were transferred into an acid fuchsin solution (0.2 M acid fuchsin and 0.8% glacial acetic acid in tap water) and heated in a microwave oven for 30 s. After cooling, roots were transferred to 40% glycerol and the number of juveniles was counted by visually inspecting the roots with a dissection microscope.

The number of egg masses per plant was counted 6 wk after inoculation by visually inspecting the roots with a dissection microscope. The natural inbred lines were screened in batches of 20 accessions, including Columbia‐0 (Col‐0) as a reference in each batch. Each inbred line was tested in at least four technical replicates. The average number of egg masses per plant, the standard error of the mean and the number of technical replicates (*n*) of each inbred line are summarized in Table S2. The data were analysed for narrow sense heritability and genome‐wide associations as described below.

To determine the susceptibility of the *bzr1‐1D*,* gsp1‐1* and *frni1‐1* mutant lines, the number of juveniles and egg masses per plant was statistically analysed using two‐way ANOVA and post‐hoc Tukey HSD test in R (v.3.0.2, http://www.r-project.org). Each line was tested in at least three independent experiments and 18 replicates per experiment. Both genotype and experiment number were used as factors to test for significance in the ANOVA.

To collect nematode‐infected roots for gene expression analysis by quantitative reverse transcription PCR (qRT‐PCR), freshly germinated 7‐d‐old seedlings were transferred to 12 cm square plates containing MS medium and placed vertically for a further 7 d at 21°C under a 16 h : 8 h, light : dark regime. Each plate contained four seedlings. Next, the seedlings were inoculated with 180 infective J2s of *M. incognita* per plant and incubated horizontally at 24°C in the dark. In parallel, seedlings in plates without juveniles were also incubated horizontally at 24°C in the dark to serve as uninfected controls. Furthermore, whole root systems of a subset of the seedlings were collected just before the inoculation with juveniles. Similarly, at 7 d after inoculation whole root systems were collected of inoculated and non‐inoculated seedlings. Root systems of 12 seedlings were aggregated to make one sample, which was snap frozen in liquid nitrogen and then stored at −80°C until further use. Three biological replicates were performed for each experiment.

### Quantitative reverse transcription PCR

Expression analysis for a gene of interest was performed on the stored root samples produced during the nematode infection study. Whole root systems were cut from aerial parts of the seedlings and snap frozen in liquid nitrogen. Total RNA was isolated from whole roots of 12 14‐d‐old plants of *gsp1‐1, bzr1‐1D, frni1‐1* and Col‐0 wild‐type. The frozen root systems were homogenized using TissueLyser (Qiagen) twice for 30 s. Total RNA was extracted from 100 mg of the homogenate with the Maxwell Plant RNA kit (Promega) using the Maxwell 16 Robot (Promega) according to the manufacturer's protocol. The amount of total RNA per sample was determined by an ND‐1000 spectrophotometer (Isogen Life Science, Utrecht, the Netherlands). First‐strand cDNA was synthesized from total RNA using the Superscript III First‐Strand synthesis system (Invitrogen) according to the manufacturer's protocol. Samples were analysed by quantitative PCR using Absolute SYBR Green Fluorescein mix (Thermo Fisher Scientific, Waltham, USA). cDNA matching *A. thaliana* elongation factor 1 alpha was amplified as a reference for constitutive expression using primers as indicated in Table S1 (Czechowski *et al*., 2004). To quantify the expression level for the gene of interest, specific gene primers were used (Table S1). For qRT‐PCR, 5 ng cDNA was used with the following conditions: 15 min at 95°C, 40 cycles of 30 s at 95°C, 30 s at 62°C and 30 s at 72°C, and a final incubation of 5 min at 72°C. The relative expression ratio between the gene of interest and the reference gene was calculated as described elsewhere (Pfaffl, 2001). This ratio was statistically analysed for significance with a two‐way ANOVA and post‐hoc Tukey HSD test in R (*P* < 0.05).

### Root phenotypes

Arabidopsis seedlings were allowed to germinate and grow for 14 d on MS medium as described above. To determine the number of root tips and root length of the seedlings, the complete plants were transferred from the media onto a plastic tray with water. Next, the leaves of the seedlings were removed and the roots were spread out over the surface of the tray. A scan of the roots was made with a photo scanner (Epson Perfection V800). The scan was analysed to measure root length using WinRhizo package for Arabidopsis (WinRhizo pro2015; Regent Instruments Inc., Ville de Québec, Canada). The number of root tips was counted by visually inspecting the scan. Differences in the number of root tips and the root length per plant were statistically analysed for significance with a two‐way ANOVA and post‐hoc Tukey HSD test in R (*P* < 0.05).

### Confocal microscopy of *BZR1‐CFP*

Seeds of the Arabidopsis *BZR1:CFP* reporter line and Col‐0 were vapour sterilized and incubated for 3 d at 4°C in the dark as described above. Next, 20 seeds were transferred to 12 cm square plates with MS media and placed vertically in a growth chamber at 21°C with a 16 h : 8 h, light : dark regime. After 5 d, the seedlings were inoculated with 25 surface‐sterilized juveniles of *M. incognita* per plant and placed vertically at 24°C in the dark. Three days after inoculation seedlings were transferred to a microscope slide and analysed with a Zeiss LSM 710 confocal microscope. Seedlings were incubated in 0.5 μg ml^−1^ propidium iodide in phosphate‐buffered saline to stain the plant cell walls. The emission spectra were set to 463–538 and 586–719 nm for cyan fluorescent protein (CFP) and propidium iodide, respectively. Non‐adjusted images were analysed with ImageJ, wherein the pixel intensity of the root area was compared to the background. Data of two independent experiments, including the analysis of 10 seedlings per experiment, were analysed with a two‐way ANOVA and post‐hoc Tukey HSD test in R. Images were enhanced in brightness for publication in print.

### Narrow‐sense heritability

To estimate the amount of variation that can be explained by genome‐wide association mapping, we calculated the narrow‐sense heritability. For this, we used a mixed model approach using efficient mixed‐model association (EMMA) based on restricted maximum likelihood (REML) to estimate the variance components as described (Kang *et al*., 2008; Rockman *et al*., 2010). The kinship matrix was calculated using all 214 051 SNPs (Horton *et al*., 2012) and narrow sense heritability was calculated as

$$h^{2}=\frac{V_{g}}{V_{g}+V_{E}}$$

where *V*_g_ is the genetic variance and *V*_E_ is the residual variance, as estimated by REML (excluding SNPs with a frequency < 0.05 from the estimation).

### Genome‐wide association mapping

Genotypic means of the egg mass data were used as input for the GWA mapping using 214 051 SNPs (Horton *et al*., 2012) using rrBLUP and the TAIR10 database (Yu *et al*., 2006; Endelman, 2011). First, a kinship matrix based on all SNPs was constructed to correct for population structure. Second, association mapping was done, excluding SNPs with a frequency < 0.05 from analysis. SNPs with a −log_10_(*P*) > 5.0 were considered significantly associated with phenotypic variance. To determine the false discovery rate at this threshold for significance, an empirical multiple testing threshold was calculated by permutation. Trait levels were randomly assigned to the genotypes, after which the association mapping was performed as described above. This procedure was repeated 1000 times resulting in a false discovery rate of 0.2 at −log_10_(*P*) > 5.0. To calculate how much of the total narrow sense heritability can be explained by significantly associating SNPs we used an additive linear model incorporating all SNPs in order to avoid a bias in SNPs capturing the same variation.

The linkage disequilibrium between SNPs was calculated using correlation analysis. First, the SNPs were converted to binary traits (either 0 or 1), which was possible because the HapMap genetic map was constructed with SNPs with only two variants per site. Per two locations the correlation between SNPs was calculated by Pearson correlation (as provided by R). The squared correlations are reported because the direction of the correlation does not confer real information (as the conversion to binary was arbitrary).

## Results

### Quantitative variation in susceptibility to *M. incognita*

**Figure 1:**
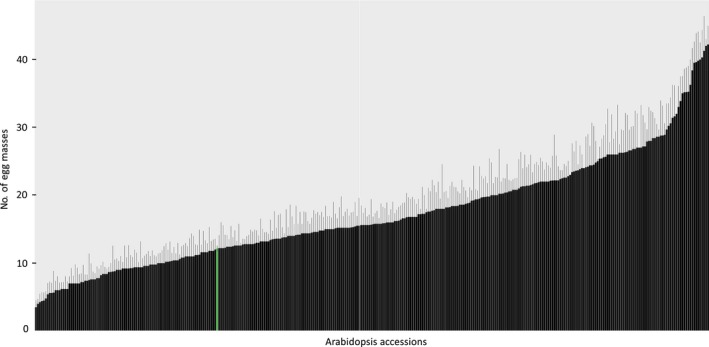
Quantitative variation in susceptibility of *Arabidopsis thaliana* to the root‐knot nematode *Meloidogyne incognita*. Average number of egg masses per plant including standard error of the mean on 340 natural inbred lines of Arabidopsis (accessions) at 6 wk after inoculation with 2^nd^ stage juveniles of *M. incognita*. The green bar indicates the number of egg masses per plant for Col‐0, which was used as a reference throughout this study.

Arabidopsis has not been systematically analysed for intraspecific variation in susceptibility to *M. incognita* before. To investigate whether Arabidopsis harbours any significant quantitative variation in susceptibility to *M. incognita*, we tested seedlings of 340 natural inbred lines of the Arabidopsis HapMap population with nematode bioassays *in vitro*. These natural inbred lines were phenotypically screened for reproductive success of *M. incognita* in batches of 20 accessions, including Col‐0 as reference in each batch. Approximately 60 accessions were tested multiple times in different batches to monitor consistency across different batches. Six weeks after inoculation, the average number of egg masses of *M. incognita* on the 340 accessions ranged from five to 45 per plant (Fig. 1; Table S2). Inoculations with *M. incognita* on Col‐0 resulted on average in 12 egg masses per plant. Based on our extensive phenotype screening we concluded that Arabidopsis harbours large quantitative variation in susceptibility to *M. incognita*.

To estimate how much of the variance in reproductive success of *M. incognita* was caused by underlying genetic variation in the Arabidopsis lines, we calculated the narrow‐sense heritability. Using 214 051 SNPs as a basis for the genetic similarity, we estimated that 52% of the variation in susceptibility to *M. incognita* was attributable to additive genetic variation between the different Arabidopsis lines. We therefore decided to use our data set to identify genome‐wide associations between SNPs in Arabidopsis and susceptibility to *M. incognita*.

### Four QTLs for susceptibility to *M. incognita* in Arabidopsis

**Figure 2:**
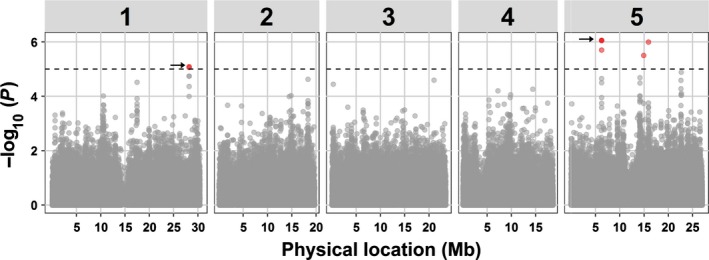
Manhattan plot of associations between 199 252 single nucleotide polymorphisms (SNPs) and the number of egg masses per plant of *Meloidogyne incognita* in Arabidopsis. Dashed horizontal line indicates threshold for significance in genome‐wide association mapping set at −log_10_(*P*) = 5. Red dots indicate the positions of eight significantly associated SNPs, of which five are overlapping and are indicated by the arrows. Numbers 1–5 in grey rectangles mark the five chromosomes of Arabidopsis.

To link allelic variation in Arabidopsis to reproductive success of *M. incognita*, we mapped genome‐wide associations underlying the number of egg masses per plant using linear mixed models (Yu *et al*., 2006; Endelman, 2011). Only SNPs with a minor allele frequency above 0.05 (199 252 SNPs) were included in the analysis. We identified significant associations between eight SNPs and the number of egg masses per plant 6 wk after inoculation with *M. incognita* in Arabidopsis (threshold for significance −log_10_(*P*) > 5; Fig. 2). Furthermore, by using an additive linear model incorporating all SNPs again, we calculated that 22% of the total variation in susceptibility of Arabidopsis can be linked to these eight SNPs.

LD in populations of natural inbred lines of Arabidopsis decays on average within 10 kb (Kim *et al*., 2007). Based on this predicted LD decay, we aggregated the eight SNPs into four QTLs located on two chromosomes (Table 1). We also analysed the specific LD between the eight significantly associated SNPs (Fig. S1). As expected, only low LD was observed between SNPs located in different QTLs (*r* ^2^ < 0.11). However, moderate LD was observed for the SNPs in QTL1 on chromosome 1 (*r* ^2^ = 0.55), while strong LD was observed for the four SNPs in QTL2 on chromosome 5 (*r* ^2^ > 0.99). The two SNPs marking QTLs 3 and 4 segregate independently (*r* ^2^ = 0.01). In conclusion, allelic variation in at least four genome locations is linked to quantitative variation in susceptibility to *M. incognita* in our population of Arabidopsis natural inbred lines.

**Table 1:** Eight single nucleotide polymorphisms (SNPs) significantly associated with reproductive success of *Meloidogyne incognita* aggregate into four quantitative trait loci (QTLs) located on two chromosomes of Arabidopsis

QTL	Chromosome	Position (bp)	SNP^a^	SNP frequency^b^	−Log_10_(*P*)^c^	Effect size	SNP located in gene
1	1	28 187 392	C : G	278 : 71	5.1	5.62	At1G75080
		28 188 151	C : G	102 : 247	5.1	5.10	At1G75090
2	5	6 263 591	A : T	139 : 210	6.1	5.31	At5G18780
		6 263 577	A : T	139 : 210	6.1	5.31	At5G18780
		6 263 644	A : T	140 : 209	5.7	5.18	At5G18780
		6 263 678	C : G	139 : 210	6.1	5.31	At5G18780
3	5	14 913 458	A : T	288 : 61	5.5	5.35	At5G37540
4	5	15 904 331	C : T	290 : 59	6	4.47	At5G39740

^a^Possible alleles for each SNP position. ^b^Frequency of lines harbouring the SNP. ^c^Level of significance of the association of an individual SNP.

**Figure 3:**
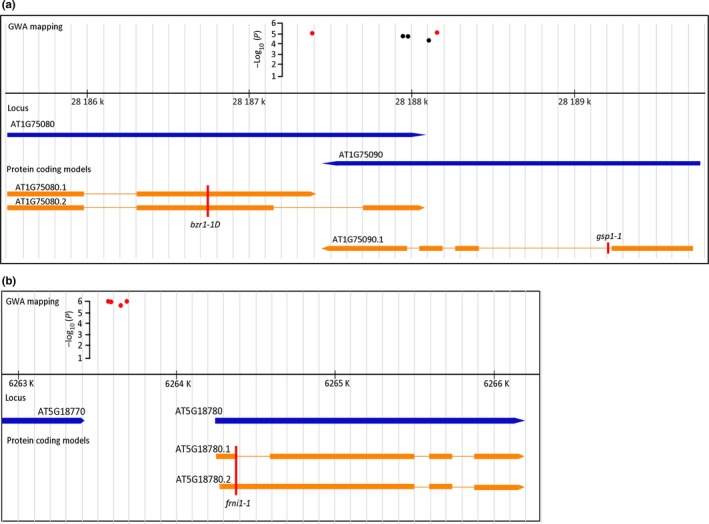
Overview of the genomic region harbouring QTL1 and QTL2 located on chromosome 1 and 5, respectively. (a) Genomic region of QTL1. The red dots represent the significantly associated single nucleotide polymorphisms (SNPs). The black dots represent three SNPs in strong linkage disequilibrium, but with −log_10_(*P*) scores below 5. The blue arrows represent two predicted genes (At1G75080 and At1G75090) in complementary orientation. Transcripts deriving from these genes are indicated in orange, with rectangles marking the protein coding exons. The red vertical line marked as *bzr1‐1D* indicates the position of the dominant ethyl methanesulfonate (EMS) mutation in the *BZR1* gene. The red vertical line marked as *gsp1‐1* indicates the position of the T‐DNA insert in a homozygous knockout mutant of *GSP1*. (b) Genomic region harbouring QTL2. The red dots represent the significantly associated SNPs. The blue arrows represent two predicted genes (At5G18770 and At5G18780) in similar orientation. Transcripts deriving from At5G18780 are indicated in orange, with rectangles marking the protein coding exons. The red vertical line marked as *frni1‐1* indicates the position of the T‐DNA insert in a homozygous knockout mutant of *FRNI1*.

To further investigate the genetic architecture underlying the reproductive success of *M. incognita* in Arabidopsis, we focused on QTL1 and QTL2 located on chromosomes 1 and 5, respectively. QTL1 is marked by two significantly associated SNPs with moderate LD (markers Chr1.28187392 and Chr1.28188151). These two SNPs were located within 1 kb distance from each other (Table 1; Fig. 3a). SNP marker Chr1.28187392 is located in *BRASSINAZOLE‐RESISTANT 1* (*BZR1*; AT1G75080) (Fig. 3a). The neighbouring SNP marker Chr1.28188151 is located in a predicted gene in complementary orientation encoding a putative DNA glycosylase superfamily protein (AT1G75090; hereafter named *GSP1*). Marker Chr1.28188151 was in strong LD (*r* ^2^ > 0.94) with three other markers at this locus (i.e. Chr1.28187959, Chr1.28187978 and Chr1.28188103), which were just below our threshold for significance in the GWA. Marker Chr1.28188103 was located in *GSP1*, while Chr1.28187959 and Chr1.28187978 were located in the regions where transcripts of *BRZ1* and *GSP1* overlap.

We used SNPs markers Chr1.28187392 and Chr1.28188151 to determine the most susceptible and the least susceptible haplotype for QTL1. Arabidopsis lines harbouring a C at Chr1.28187392 (*n *=* *278) were less susceptible to *M. incognita* than those harbouring a G (*n *=* *71). Similarly, lines harbouring a G at Chr1.28188151 (*n *=* *247) were also less susceptible than those harbouring a C (*n *=* *102). These polymorphisms occurred in four haplotype combinations: CC (*n *=* *32), GC (*n *=* *62), CG (*n *=* *232) and GG (*n *=* *2). Interestingly, lines with the most prevalent CG haplotype (e.g. Col‐0) were also the least susceptible to *M. incognita*, which could point to a selective advantage of this haplotype (Fig. S2).

Next, we focused on four significantly associated SNP markers with strong LD (i.e. Chr5.6263591, Chr5.6263577, Chr5.6263644 and Chr5.6263678) that mark QTL2 on chromosome 5. The SNPs are all located in an intergenic region *c*. 600 bp upstream of predicted gene At5G18780 (Fig. 3b). Two splice variants have been observed for At5G18780, both with unknown function. The protein encoded by At5G18780 is annotated as F‐box/Ribonuclease inhibitor‐like superfamily protein of 441 amino acids (hereafter *FRNI1*). The predicted topology of *FRNI1* includes an amino terminal F‐box of 50 amino acids long (pfam 00646), seven leucine‐rich repeats with similarity to ribonuclease inhibitor 1 (RNI) and a carboxy terminal FBD domain (pfam08384) that is found in F‐box domain‐containing plant proteins.

### *BZR1* and *FRNI1* (co‐)regulate reproductive success of *M. incognita*

**Figure 4:**
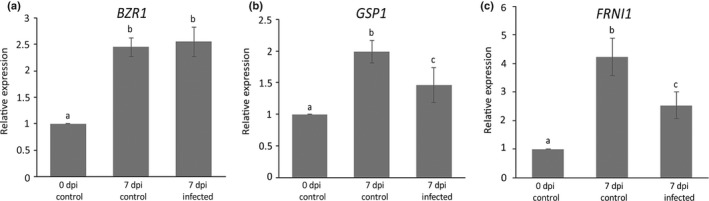
Relative expression of *BZR1*,*GSP1* and *FRNI1* in infected and noninfected roots of wild‐type Arabidopsis Col‐0 plants at 7 d after inoculation (dpi) with *Meloidogyne incognita*. The expression levels of (a) *BZR1*, (b) *GSP1* and (c) *FRNI1* are given as ratios relative to the expression levels of these genes at the time of inoculation. Data reflect gene expression levels in whole roots collected at the time of inoculation with *M. incognita* (0 dpi control), in whole roots collected at 7 d after mock‐inoculation (7 dpi control) and 7 d after inoculation with *M. incognita* (7 dpi infected). Bars represent average values based on three independent biological samples with three technical replicates per biological sample. Error bars represent ±SEM. Different lower‐case letters indicate statistical difference determined by ANOVA with post‐hoc Tukey HSD (*P* < 0.05).

To find further support for a role of *BZR1*,* GSP1* and *FRNI1* in susceptibility of Arabidopsis to *M. incognita*, we first assessed their expression levels in roots of infected and non‐infected seedlings using qRT‐PCR. Expression of the genes was determined in whole root systems collected at the time of inoculation and at 7 d after inoculation in infected and non‐infected plants. This set up allowed us to study the developmental regulation of the genes in young Arabidopsis seedlings, as well as their regulation in response to infection by *M. incognita*. *BZR1*,* GSP1* and *FRNI1* were all upregulated in non‐infected roots of Arabidopsis seedlings, as they developed in the 7 d after the time of inoculation (Fig. 4). Expression of both *GSP1* and *FRNI1* was significantly downregulated in nematode‐infected roots at the same time point after inoculation (*P* < 0.05). By contrast, infection by *M. incognita* did not alter the developmentally regulated expression of *BZR1*. As we used whole root systems, dilution effects could keep local changes in expression of *BZR1* at the infection site below the detection limits of the qRT‐PCR. To address this concern, we also investigated the expression of *BZR1* with confocal microscopy of the Arabidopsis BZR1:CFP reporter line at 3 d after inoculation with *M. incognita* (Fig. S3). Based on image analysis, we concluded that infections with *M. incognita* do not lead to significant changes in *BZR1* expression at the infection site of the nematodes.

**Figure 5:**
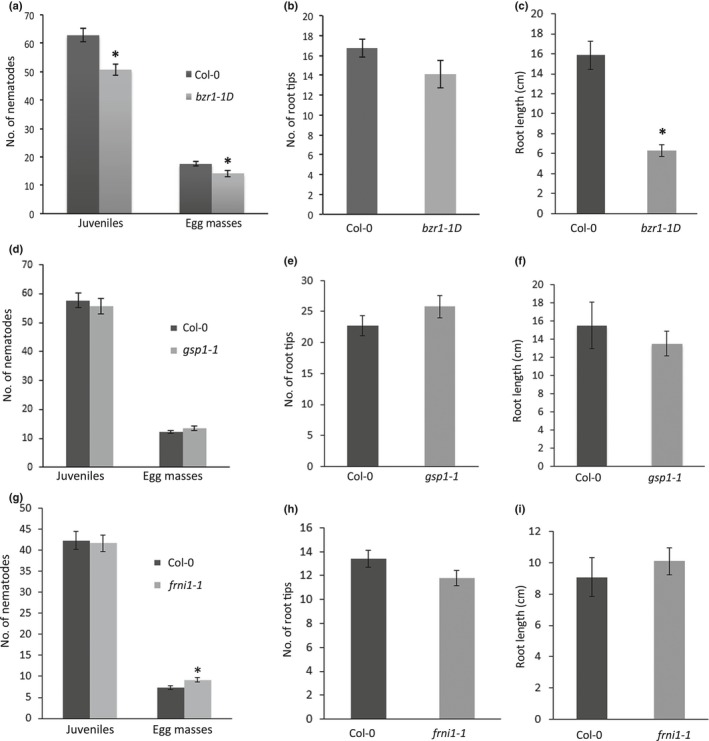
Susceptibility of a dominant positive ethyl methanesulfonate (EMS) mutant *bzr1‐1D* and homozygous T‐DNA insert mutants *gsp1‐1* and *frni1‐1* of Arabidopsis to *Meloidogyne incognita*. (a) Number of juveniles at 7 d after inoculation and egg masses per plant at 6 wk after inoculation on *bzr1‐1D* and wild‐type Arabidopsis plants. (b) Number of root tips and (c) total root length of seedlings of *bzr1‐1D* at the age of inoculation. (d) Number of juveniles at 7 d after inoculation and egg masses per plant at 6 wk after inoculation on *gsp1‐1* and wild‐type Arabidopsis plants. (e) Number of root tips and (f) total root length of seedlings of *gsp1‐1* at the age of inoculation. (g) Number of juveniles at 7 d after inoculation and egg masses per plant at 6 wk after inoculation on *frni1‐1* and wild‐type Arabidopsis plants. (h) Number of root tips and (i) total root length of seedlings of *frni1‐1* at the age of inoculation. (a, d, g) Bars reflect the averages and SEM of three independent experiments (*n *>* *50). (b, c, e, f, h, i) Bars represent the mean ± SEM of three independent experiments (*n *>* *12). Data were statistically tested for significance with ANOVA with post‐hoc Tukey HSD: *, *P* < 0.05.

To test if *BZR1*,* GSP1* and *FRNI1* are required for reproductive success of *M. incognita*, we challenged several Arabidopsis mutant lines with infective juveniles in a bioassay. Homozygous Arabidopsis T‐DNA knockout mutants of *BZR1* have a lethal phenotype and could not be used to test the involvement of this gene in the reproductive success of *M. incognita*. Instead, we analysed the susceptibility of the dominant positive EMS mutant Arabidopsis line *bzr1‐1D* in our bioassays with *M. incognita* (Wang *et al*., 2002). Both the number of J2s of *M. incognita* per plant at 7 d after inoculation and the number of egg masses per plant at 6 wk after inoculation were significantly reduced on the *bzr1‐1D* mutant line compared to the wild‐type Arabidopsis plants (Fig. 5a). The *bzr1‐1D* mutant harbours a functional mutant allele of the *BZR1* transcription factor that makes it insensitive to the brassinosteroid (BR) biosynthetic inhibitor brassinazole (Wang *et al*., 2002). Seedlings of the *bzr1‐1D* mutant typically show anomalous root architecture under specific light conditions (Wang *et al*., 2002), and this could affect susceptibility to nematode infections. Indeed, in our experimental set‐up the average total root length at the time of inoculation was significantly smaller in *bzr1‐1D* mutants as compared to wild‐type Col‐0 plants (Fig. 5c). More importantly, susceptibility of plants to root‐knot nematodes is known to depend on the number of available root tips at the time of inoculation. This parameter of root architecture was not significantly different between the *bzr1‐1D* mutant and the wild‐type Arabidopsis plants (Fig. 5b).

A homozygous Arabidopsis T‐DNA mutant line was available for the *GSP1* gene, which harbours an insert in the predicted first intron of the coding sequence of *GSP1* (Fig. 3a). Expression of *GSP1* was strongly reduced in roots of the *gsp1‐1* mutant line, but not completely knocked‐out (Fig. S4a,b). Despite this reduction in gene expression, the number of egg masses per plant at 6 wk after inoculation was not significantly different in the *gsp1‐1* mutant line, when compared to wild‐type plants (Fig. 5d). Similarly, the number of infective juveniles per plant at 7 d after inoculation was also not significantly different between the *gsp1‐1* mutant and wild‐type plants. Furthermore, the root architecture of this mutant was not significantly different from wild‐type Arabidopsis plants (Fig. 5e,f).

**Figure 6:**
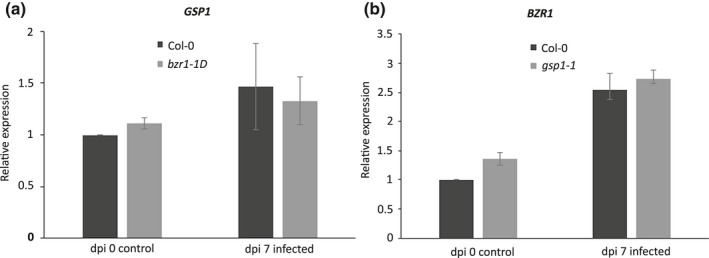
Mutation in the Arabidopsis *bzr1‐1D* line does not affect expression of neighbouring *GSP1*, and vice versa. (a) Relative expression of *GSP1* in *bzr1‐1D* and wild‐type Arabidopsis plants at 7 d after inoculation (dpi) with *Meloidogyne incognita*. (b) Relative expression of *BZR1* in *gsp1‐1* and wild‐type Arabidopsis plants at 7 d after inoculation with *M. incognita*. Gene expression levels are determined by quantitative reverse transcription PCR and presented here as a ratio relative to the expression level in wild‐type Arabidopsis plants at the time of inoculation. Mars represent average values based on three independent biological samples with three technical replicates per biological sample. Error bars represent ± SEM.

*BZR1* and *GSP1* are located in antisense direction and their coding sequences partially overlap. *BZR1* and *GSP1* could therefore act as *cis*‐natural antisense pairs, which could lead to the formation of siRNA and thus transcript breakdown. To test this, we analysed the expression of *GSP1* in *bzr1‐1D* and the expression of *BZR1* in *gsp1‐1*. In a comparison with wild‐type Arabidopsis seedlings at 7 d after inoculation, the expression of *GSP1* was not altered by the EMS mutation in *bzr1‐1d*, and vice versa the expression of *BZR1* was not altered by the T‐DNA insert in *gsp1‐1* (Fig. 6). Our data therefore showed that it is unlikely that the phenotype of the *bzr1‐1D* mutation arises through it actions on transcript levels of *GSP1*.

A homozygous Arabidopsis knock‐out line was also available for the *FRNI1* gene, which harbours a T‐DNA insert in the first predicted exon of *FRNI1* (Fig. 3b). qRT‐PCR showed that the expression of *FRNI1* was completely knocked‐out in roots of the *frni1‐1* mutant line (Fig. S4c,d). The number of egg masses on roots of the *frni1‐1* mutant line at 6 wk after inoculation was significantly higher as compared to the wild‐type Arabidopsis plants (Fig. 5g). By contrast, we observed no significant difference in the number of juveniles inside roots at 7 d after inoculation between *frni1‐1* and wild‐type Arabidopsis plants. The root architecture of the *frni1‐1* mutant was also not significantly different from wild‐type Arabidopsis plants (Fig. 5h,i). Altogether, we concluded that *BZR1* and *FRNI* probably function as (co‐)regulators of reproductive success of *M. incognita* in Arabidopsis. Allelic variation in these genes may therefore contribute to quantitative variation in susceptibility to *M. incognita* in our collection of Arabidopsis lines. By contrast, despite its downregulation in association with nematode infections, allelic variation in *GSP1* is less likely to be causal for quantitative variation in susceptibility of Arabidopsis to *M. incognita*.

**Figure 7:**
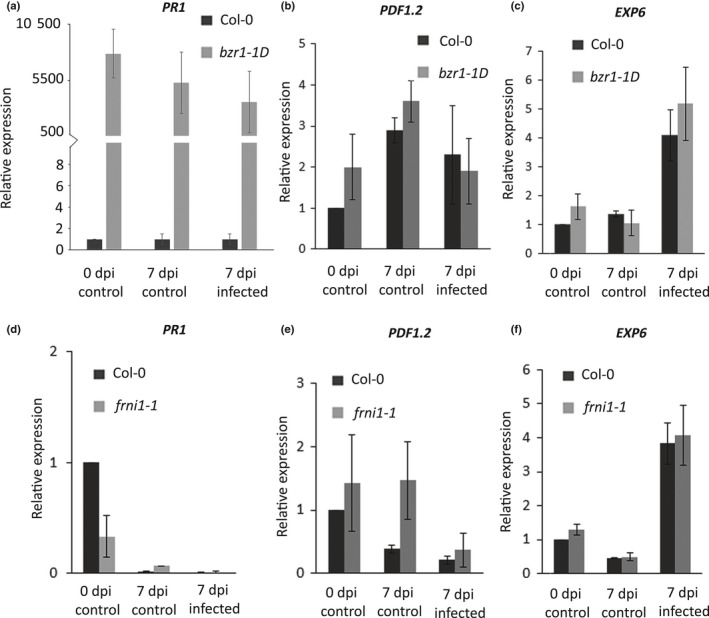
Differential expression of marker genes for salicylic acid‐ (*PR1*) and jasmonic acid‐ (*PDF1.2*) dependent defence responses and plant cell expansion (*EXP6*) in the *bzr1‐1D* and *frni1‐1* mutant and wild‐type Arabidopsis plants. Relative expression levels of (a) *PR1*, (b) *PDF1.2* and (c) *EXP6* in *bzr1‐1D* and wild‐type Arabidopsis plants. Relative expression levels of (d) *PR1*, (e) *PDF1.2* and (f) *EXP6* in *gsp1‐1* and wild‐type Arabidopsis plants. Gene expression levels are determined with quantitative reverse transcription PCR and presented here as a ratio relative to the expression level in wild‐type Arabidopsis plants at the time of inoculation. Data reflect expression in whole roots collected at the time of inoculation with *Meloidogyne incognita* (0 d post‐inoculation (dpi) control), in whole roots collected at 7 d after mock‐inoculation (7 dpi control) and 7 d after inoculation with *M. incognita* (7 dpi infected). Bars represent average values based on three independent biological samples with three technical replicates per biological sample. Error bars indicate SEM.

BZR1 is a master regulator of both cell proliferation, differentiation and defence. As such it regulates cell elongation, which is evident from the reduced root growth phenotype of the *bzr1‐1D* mutant. Reduced cell growth may affect the expansion of nematode‐induced giant cells, but we could not exclude the possibility that the *bzr1‐1D* mutant is also affected in its ability to mount a defence response to *M. incognita*. We therefore analysed the expression of markers for salicylic acid‐ and jasmonic acid‐related defence responses (i.e. At2G14610 (*PR1*) and A5G44120 (*PDF1.2*)) and a marker for cellular expansion (i.e. At2G28950 (*EXP6*)) in nematode‐infected roots of the *bzr1‐1D* mutant line and wild‐type Arabidopsis. Surprisingly, the expression of *PR1* was constitutively and highly upregulated at the time of inoculation and at 7 d after inoculation in both infected and non‐infected *bzr1‐1D* mutants when compared to wild‐type Arabidopsis plants (Fig. 7a). By contrast, the marker genes for jasmonic acid‐dependent defences and cellular expansion were not differentially regulated in *bzr1‐1D* and wild‐type Arabidopsis (Fig. 7b,c). The function of FRNI1 is not known, and we therefore conducted a similar marker gene experiment on nematode‐infected roots of the *frni1‐1* mutant line. However, none of the marker genes was differentially regulated between *frni1‐1* and wild‐type Arabidopsis plants (Fig. 7d–f).

## Discussion

Most genetic analyses of wild relatives of crop plant species have focused on identifying new sources of dominant resistance to nematodes, while largely disregarding natural variation in susceptibility. Here, we used GWA mapping to assess the genetic underpinnings of a large variation in susceptibility to the root‐knot nematode *M. incognita* in a population of natural inbred lines of Arabidopsi*s*. By applying a −log_10_(*P*) score of 5 as a threshold for significance (corresponding to a false discovery rate of 0.2), we identified four QTLs in Arabidopsis associated with the number of egg masses of *M. incognita* at 6 wk post‐inoculation (Fig. 2). These four QTLs probably harbour allelic variants that are causally related to reproductive success of *M. incognita* on Arabidopsis.

The ability of Arabidopsis to support reproduction of root‐knot nematodes is thought to be a complex trait involving many different plant genes (Jammes *et al*., 2005; Dubreuil *et al*., 2007; Barcala *et al*., 2010; Portillo *et al*., 2013; Cabrera *et al*., 2014). Given the anticipated complexity of this trait, the number of QTLs significantly associated with reproduction of *M. incognita* in our GWA study was relatively small. It is possible that the genetic architecture underlying susceptibility of Arabidopsis to *M. incognita* is much simpler than previously thought. However, it should be noted that only 22% of the observed phenotypic variance can be linked to the four QTLs identified our GWA study. This could indicate the presence of an abundance of rare alleles (with minor allele frequencies below 0.05), which are capturing most of the variation but were excluded from GWA mapping. Alternatively, many QTLs for susceptibility to *M. incognita* in the Arabidopsis genome may have small effect sizes that cannot be detected with the resolution of our bioassays. In either case, our GWA study probably underestimates the complexity of the genetic architecture of susceptibility to *M. incognita* in Arabidopsis.

Others have investigated the genetic architecture of responses to biotic stresses in Arabidopsis by accepting a less stringent threshold for significant associations in GWA mapping (e.g. −log_10_(*P*) > 4; El‐Soda *et al*., 2015; Kloth *et al*., 2016; Kooke *et al*., 2016; Davila Olivas *et al*., 2017). Similarly relaxing the stringency in our analysis would result in significant associations between 36 SNPs (located within 19 QTLs) and reproduction of *M. incognita* on Arabidopsis. Lowering the threshold for significance in the GWA mapping may thus reveal more common alleles with smaller effect sizes in our population of Arabidopsis lines (Korte & Farlow, 2013; Kooke *et al*., 2016). However, this would also raise the false discovery rate to 60%, which would reduce the chances of identifying causal genes in follow‐up studies.

To assess whether our GWA study (using stringent criteria) can help to identify genes involved in susceptibility of Arabidopsis to *M. incognita*, we focused on two SNPs located in QTL1 on chromosome 1 (Fig. 3). Chr1.28187392 showed moderate LD (*r* ^2 ^= 0.55) with Ch1.28188151, while LD seems to rapidly decay with SNPs directly flanking Chr1.28187392 and Chr1.28188151. Based on the locations of the two significant SNPs and the predicted LD decay in this region, we concluded that *BZR1* and *GSP1* were the only two candidates in this region that could contribute to the variance in susceptibility of Arabidopsis to *M. incognita*.

Our infection assays with the dominant positive *bzr1‐1D* mutant line showed that BZR1 probably acts as a rate‐limiting factor in the reproductive success of *M. incognita* in Arabidopsis (Fig. 5). The number of juveniles inside seedlings during the early stages of parasitism and the number of egg masses at 6 wk post‐inoculation was consistently smaller on the *bzr1‐1D* mutant when compared to wild‐type Col‐0. BZR1 is constitutively active in the *bzr1‐1D* mutant line, which simulates the accumulation of BR (Wang *et al*., 2002). Under specific light conditions the dominant *bzr1‐1D* mutation results in an anomalous root architecture. In our nematode infection assays the number of root tips at the time of inoculation was not significantly different between the *bzr1‐1D* mutant line and wild‐type Col‐0. This is important because the invasion of Arabidopsis by *M. incognita* occurs only in the transition zone close to root tips (Sijmons *et al*., 1991). However, the reduced total root length of the *bzr1‐1D* mutant could point to defects in cell growth, which may affect the expansion of nematode‐induced giant cells.

The transcription factor BZR1 is at the end of a signalling cascade which is activated by the BRI1/BAK1 co‐receptor complex upon detection of brassinolide (Jaillais & Vert, 2016). The activation of BR signalling in Arabidopsis results in the dephosphorylation and translocation to the nucleus of BZR1, where it binds to DNA and specifically activates or represses the expression of almost 1000 genes (Sun *et al*., 2010; Di Rubbo *et al*., 2011). BR signalling plays a crucial role in determining cell growth by promoting elongation of differentiated cells, but also by regulating the transition between cell cycle progression and cell differentiation (Jaillais & Vert, 2016). Aberrant progression through the mitotic cell cycle, extensive cell elongation and expansion are all considered essential steps in the ontogeny of giant cells in nematode‐infected roots of *A. thaliana* (de Almeida Engler & Gheysen, 2013; Kyndt *et al*., 2013; Vieira *et al*., 2013). The outcome of BR signalling in roots is cell type and position specific, but generally antagonises the effect of auxin (Chaiwanon & Wang, 2015). BZR1 regulates the expression of several genes related to auxin biosynthesis and signalling (Sun *et al*., 2010). For instance, BZR1 directly represses the expression of PIN auxin efflux carriers involved in directing polar auxin transport towards root tips (i.e. *PIN3* and *PIN4*; Feraru & Friml, 2008; Sun *et al*., 2010; Vragović *et al*., 2015). Recently, it was shown that development of *M. incognita* is hampered on Arabidopsis knockout mutants of *PIN3* and *PIN4* (Kyndt *et al*., 2016). PIN3 and PIN4 are thought to be involved in redirecting the flow of auxin during giant cell formation. Similarly, BZR1 regulates the expression of genes involved in plant cell wall plasticity, which is a fundamental requirement for cell growth but also for the expansion of giant cells (e.g. *EXPA1*; Jammes *et al*., 2005; Sun *et al*., 2010).

Furthermore, BZR1 also regulates the trade‐off between growth and immunity, which may explain the constitutive upregulation of *PR1* that we observed in the *bzr1‐1D* mutant (Lozano‐Duran *et al*., 2013; Lozano‐Durán & Zipfel, 2015). The loss of susceptibility to *M. incognita* in the *bzr1‐1D* mutant could therefore also reflect alterations in basal immunity of Arabidopsis seedlings. This latter scenario would be in agreement with the recently observed enhanced resistance of transgenic *Lotus japonicus* plants ectopically expressing the Arabidopsis *bzr1‐1D* allele to feeding by onion thrips (Miyaji *et al*., 2014). Altogether, we conclude that BZR1 probably (co‐)regulates susceptibility to *M. incognita* in *A. thaliana* through its role in plant cell growth, basal defence or both. Allelic variation in *BZR1* could therefore be casual for some of the observed variance in reproductive success of *M. incognita* in our population of Arabidopsis lines.

The second SNP marker significantly associated with the number of egg masses of *M. incognita* per plant in QTL1 was located in the first exon of *GSP1*, a putative DNA glycosylase superfamily protein. The function of *GSP1* has not been studied before, but based on sequence homology it is predicted to be involved in base‐excision repair of DNA (Manova & Gruszka, 2015). Despite the fact that *GSP1* is strongly downregulated upon infection by *M. incognita* at 7 d after inoculation, the homozygous knockdown mutant Arabidopsis line of *GSP1* in the Col‐0 background showed no altered susceptibility to *M. incognita*. This suggests that *GSP1* is regulated in association with, but not required for, reproduction of *M. incognita* in Arabidopsis. However, it should be noted that the Arabidopsis Col‐0 line carries the nonsusceptible haplotype (i.e. CG) for this locus, and that a knockdown by the T‐DNA insert in *gsp1‐1* may therefore not lead to further reduction in susceptibility. In conclusion, we have found no evidence suggesting that allelic variation in *GSP1* significantly contributes to the variance in susceptibility of Arabidopsis to *M. incognita*.

Four co‐segregating SNPs marking QTL2 on chromosome 5 pointed at FRNI1 as a co‐regulator of susceptibility of Arabidopsis to *M. incognita*. The SNP markers are located in the putative regulatory region upstream of the predicted coding sequence of *FRNI1*, where they might affect expression levels of this gene. Unlike *BZR1*,* FRNI1* is strongly downregulated in nematode‐infected roots of wild‐type Arabidopsis Col‐0 plants. Alterations in *FRNI1* expression are therefore likely to affect the susceptibility of Arabidopsis to *M. incognita*. In fact, our data showed that the compete loss of *FRNI1* expression in the *frni1‐1* knockout mutant resulted in a small but significant increase in the number of egg masses per plant. So far, no function has been ascribed to FRNI1, but its architecture as an F‐box‐like and RNI‐like protein suggests that it might be involved in protein–protein interactions. More specifically, the F‐box is defined as a component of the E3 ubiquitin ligase complex SCF (Skp1‐cullin‐F‐box protein ligase), which targets proteins to the 26S proteasome for degradation (Lechner *et al*., 2006). F‐box‐containing proteins are involved in many cellular process in plants, including hormone signalling and defence responses. The lack of differential expression of *PR1*,* PDF1.2* and *EXP6* in nematode‐infected roots of *frni1‐1* mutant and wild‐type Arabidopsis plants offered no clue as to whether *FRNI1* co‐regulates susceptibility by affecting defence, development or both. Further investigations are therefore needed to shed light on the function of FRNI1 in Arabidopsis.

The main objective of this study was to explore the natural variation in susceptibility to *M. incognita* of Arabidopsis, which is thought to lack dominant resistance genes to this nematode species. In our phenotype screening of the Arabidopsis lines we observed an unexpected large variation in reproductive success of *M. incognita*. Extensive variation in susceptibility was also observed within a smaller set of 45 Arabidopsis inbred lines challenged with the northern root‐knot nematode *Meloidogyne hapla* (Boiteux *et al*., 1999). As the natural distribution of *M. hapla* and *A. thaliana* in temperate regions may have overlapped, this variation could be partly based on segregating major resistance genes. We found no evidence by GWA mapping that the large phenotypic variance in susceptibility to *M. incognita* is based on the presence of segregating dominant resistance genes linked to any of the QTLs. By contrast, GWA mapping of susceptibility to *Meloidogyne graminicola* in rice cultivars identified 11 genomic loci, at least one of which harbours major resistance gene homologues (Dimkpa *et al*., 2016). Our data thus indicate that plants could be made more resistant to infections of root‐knot nematodes by selecting unfavourable alleles of *S*‐genes that are essential for giant cell initiation, expansion and maintenance. However, it remains to be investigated if these loss‐of‐susceptibility alleles can be exploited by plant breeders to improve the resilience of crops without experiencing undesirable pleiotropic effects on other agronomically important traits.

## Author contributions

J.B., A.G., G.S., M.D., J.H. and S.W. conceived and designed the experiments. S.W., M.G.S., M.E.P.O., C.v.S., O.C.A.S. and J.L.L‐T. performed the experiments. S.W., M.G.S., J.L.L‐T., J.E.K., A.G., J.B. and G.S. analysed the data. S.W., M.G.S., J.E.K., A.G., J.B. and G.S. wrote the article. All authors edited and approved the final article.

## Supporting information

Please note: Wiley Blackwell are not responsible for the content or functionality of any Supporting Information supplied by the authors. Any queries (other than missing material) should be directed to the *New Phytologist* Central Office.

**Fig. S1** Linkage disequilibrium (LD) between eight SNPs in Arabidopsis significantly associated with the number of egg masses of *Meloidogyne incognita* per plant.
**Fig. S2** Haplotype‐specific susceptibility of Arabidopsis to *Meloidogyne incognita*.
**Fig. S3** Expression of *BZR1:CFP* fusion protein under the control of the endogenous *BZR1* promoter sequence in roots of Arabidopsis seedlings infected with *Meloidogyne incognita*.
**Fig. S4** Strongly reduced expression of *GSP1* and *FRNI1* in the homozygous knockout Arabidopsis line *gsp1‐1* and *frni1‐1,* respectively.
**Table S1** Primers used for RT‐PCR
**Table S2** Number of egg masses of *Meloidogyne incognita* per plant on 340 natural inbred lines of *Arabidopsis thaliana* at 6 wk after inoculation
