# Supplementary material for: Genome‐wide association mapping of the architecture of susceptibility to the root‐knot nematode Meloidogyne incognita in Arabidopsis thaliana
Source: New Phytol. 2018 Feb 22;218(2):724–37. doi: 10.1111/nph.15034 (PMC6079644; doi:10.1111/nph.15034)
Supplement: Supplementary file 1 — Fig. S1 Linkage disequilibrium (LD) between eight SNPs in Arabidopsis significantly associated with the number of egg masses of Meloidogyne incognita per plant. Fig. S2 Haplotype‐specific susceptibility of Arabidopsis to Meloidogyne incognita. Fig. S3 Expression of BZR1:CFP fusion protein under the control of the endogenous BZR1 promoter sequence in roots of Arabidopsis seedlings infected with Meloidogyne incognita. Fig. S4 Strongly reduced expression of GSP1 and FRNI1 in the homozygous knockout Arabidopsis line gsp1‐1 and frni1‐1, respectively. Table S1 Primers used for RT‐PCR Table S2 Number of egg masses of Meloidogyne incognita per plant on 340 natural inbred lines of Arabidopsis thaliana at 6 wk after inoculation [file NPH-218-724-s001.pdf]

***New Phytologist* Supporting Information**

Article title: **Genome-wide association mapping of susceptibility of *Arabidopsis thaliana* to *Meloidogyne incognita***

Authors: Sonja Warmerdam, Mark G. Sterken, Casper van Schaik, Marian E. P. Oortwijn, Octavina C. A. Sukarta, Jose L. Lozano-Torres, Marcel Dicke, Johannes Helder, Jan E. Kammenga, Aska Goverse, Jaap Bakker, Geert Smant.

Article acceptance date: 08 January 2018

The following Supporting Information is available for this article:

Fig. S1 Linkage disequilibrium (LD) between eight SNPs in Arabidopsis significantly associated with the number of egg masses of M. incognita per plant.

Fig. S2 Haplotype specific susceptibility of Arabidopsis to *M. incognita*.

Fig. S3 Expression of *BZR1:CFP* fusion protein under the control of the endogenous *BZR1* promoter sequence in roots of Arabidopsis seedlings infected with *M. incognita*.

Fig. S4 Strongly reduced expression of *GSP1 and FRNI1* in the homozygous knock-out Arabidopsis line *gsp1-1 and frni1-1,* respectively.

Table S1 Primers used for RT-PCR

Table S2 Number of egg masses of *M. incognita* per plant on 340 natural inbred lines of *Arabidopsis thaliana* at 6 weeks after inoculation.

Fig. S1 Linkage disequilibrium (LD) between eight SNPs in Arabidopsis significantly associated with the number of egg masses of *M. incognita* per plant. The LD is calculated as correlation between the eight SNPs, where a darker colour reflects higher correlations and thus a stronger LD. SNPs are indicated with the chromosome number and the location in base pairs. The legend identifies the number of counts for each correlation (r^2).

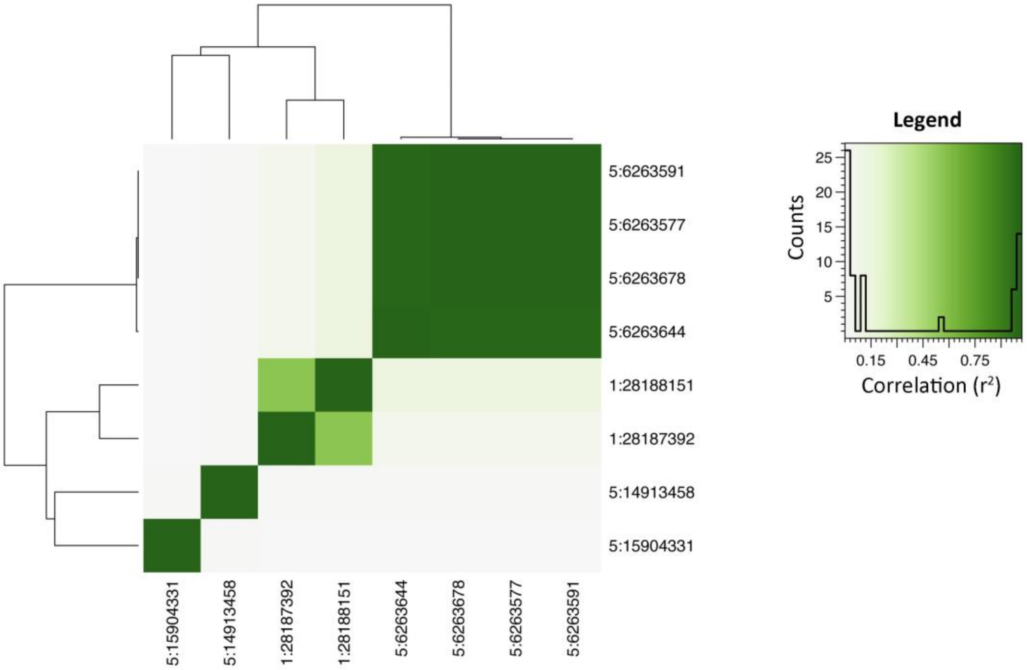

Fig. S2 Haplotype specific susceptibility of Arabidopsis to *M. incognita***.** Box plots of the number of egg masses of *M. incognita* per plant on Arabidopsis lines by haplotype at positions 28187392 and 28188151 on chromosome 1. SNP Ch1.28187392 and SNP Chr1. 28188151 are represented by the numbers 7392 and 8151, respectively. Each dot represents the average number of egg masses per plant of one accession harbouring either the haplotype CC, GC, CG, or GG.

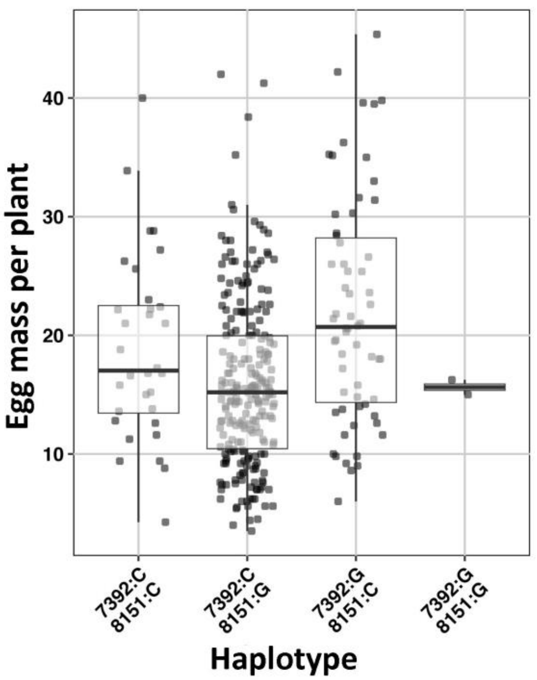

Fig S3: Expression of *BZR1:CFP* fusion protein under the control of the endogenous *BZR1* promoter sequence in roots of Arabidopsis seedlings infected with *M. incognita*. (a) Microscopic images of root tips of *BZR1:CFP* reporter lines uninfected (upper panel) and infected with *M. incognita* 3 days post inoculation. (b) Intensity levels of total CFP fluorescence based on grey value per pixel of the root area measured with ImageJ. Error bars represent standard error of the mean. Data was further analysed with ANOVA and post-hoc Tukey HSD ($p<0.05$, $n>10$).

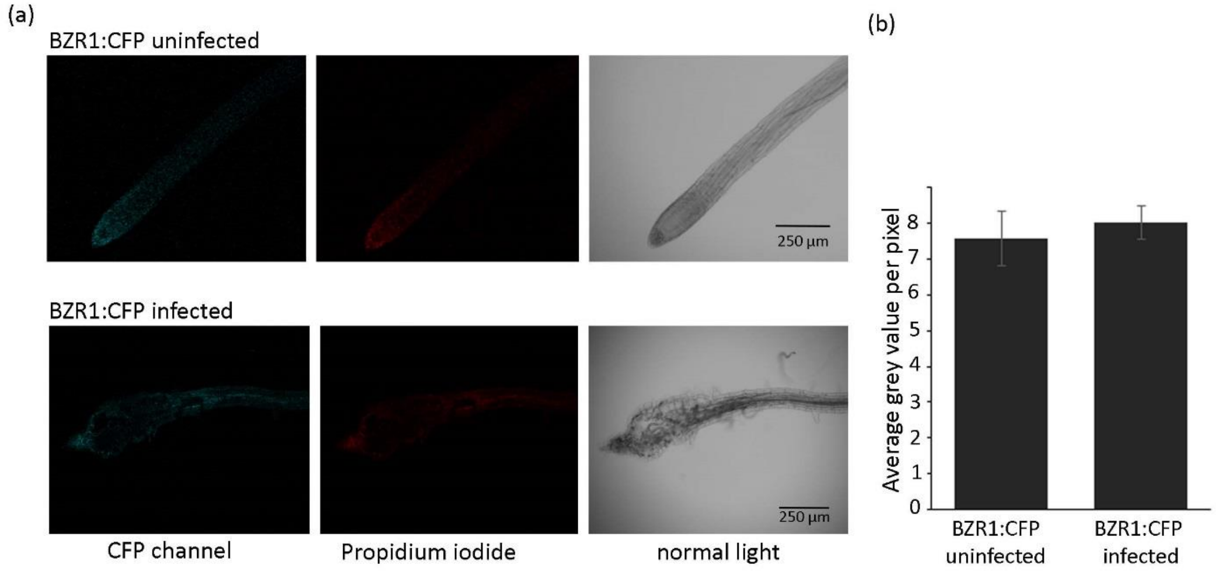

Fig. S4 Strongly reduced expression of *GSP1 and FRNI1* in the homozygous knock-out Arabidopsis line *gsp1-1 and frni1-1,* respectively. (a) Allele specific PCR on genomic DNA isolated from the Arabidopsis mutant line *gsp1-1*. PCR amplification products using primer combinations for the wildtype *GSP1* allele and for the *gsp1-1* allele including the T-DNA insert. (b) Relative gene expression of *GSP1* in the T-DNA insertion mutant *gsp1-1* as compared to the wildtype Col-0 using quantitative RT-PCR. (c) Allele specific PCR on genomic DNA isolated from the Arabidopsis mutant line *frni1-1*. PCR amplification products using primer combinations for the wildtype *FRNI1* allele and for the *frni1-1* allele including the T-DNA insert. (d) Relative gene expression of *FRNI1* in the T-DNA insertion mutant *frni1-1* as compared to the wildtype Col-0 using quantitative RT-PCR. Error bars represent standard error of the mean.

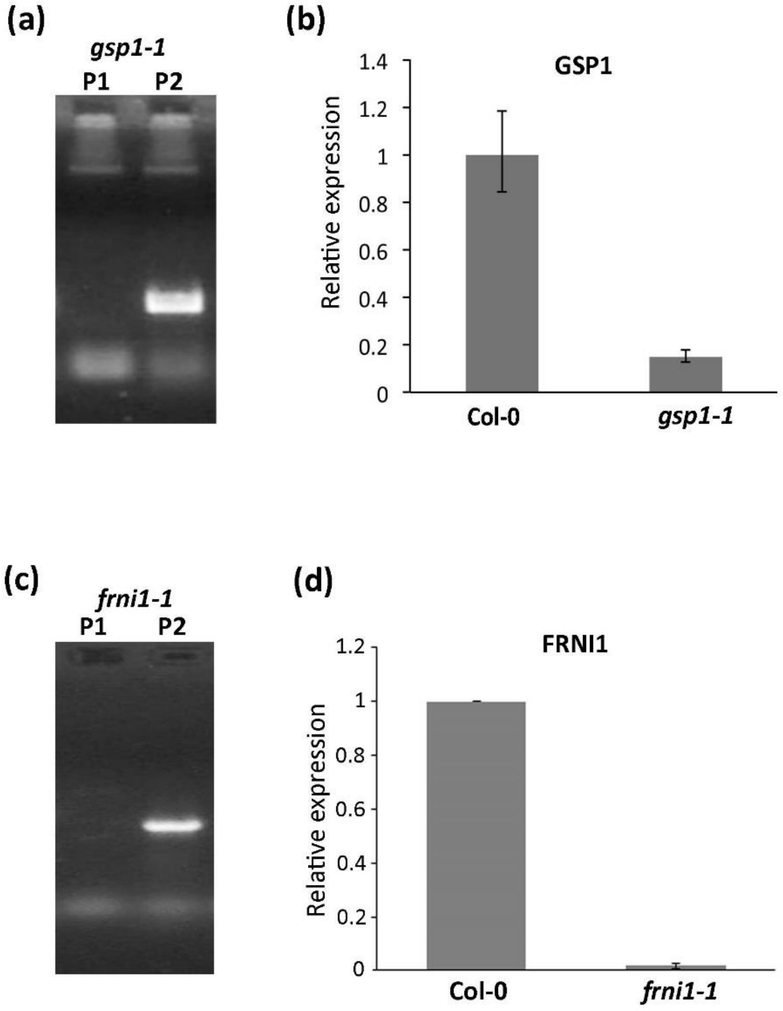

Table S1: Primers used for RT-PCR**.** Identifier mentions the corresponding gene with the forward and reverse primer sequence

Identifier	Forward	Reverse
**Expression of**		
GSP1	AACGTTCGTCAGTGAACACG	GATCGGGTCTGAATTAGGTGTG
BZR1	AACCCGAAACCGTTGCCTAA	GGGTATGAAACTGGTGGCGA
FRNI1	AGCAACGGAACTGAAACTGG	ATGTGTACTGCATCAACAACG
ELF1a	GAGTACCCACCTTTGGGACG	TTGGGTCCTTCTTGTCCACG
PR1	ACGGGGAAAACTTAGCCTGG	TTGGCACATCCGAGTCTCAC
PDF1.2	CACCCTTATCTTCGCTGCTC	GTTGCATGATCCATGTTTGG

EXP6	TGCCCATTTCTCCCAATCTCT	AACCAAGCCCAACATTGCC
**T-DNA insert**		
gsp1 wildtype	TGCTAGGTGCAATTAATGTGTTG	AGCTTCGTTCTCCGGTTAAAC
gsp1-1 allele	ATTTTGCCGATTTCGGAAC	AGCTTCGTTCTCCGGTTAAAC
frni1 wildtype	GCAATTAGCACCTCTGCAGAC	CTCTGAATCTCCAAGACGCTC
frni1-1 allele	ATTTTGCCGATTTCGGAAC	CTCTGAATCTCCAAGACGCTC

Table S2 Number of egg masses of *M. incognita* per plant on 340 natural inbred lines of *Arabidopsis thaliana* at 6 weeks after inoculation. The numbers are average values, corresponding standard error of mean, and total number of individual plants (n).

Genotype	Average number of egg masses	SE	n	Genotype	Average number of egg masses	SE	n
CS28099	3.5	1.0	4	CS76155	15.8	1.1	4
CS76206	4.0	0.7	5	CS28200	15.8	1.6	5
CS76124	4.3	1.3	4	CS76174	15.8	1.9	5
CS76179	4.4	1.3	5	CS76192	15.8	0.9	5
CS76181	4.5	1.2	4	CS76289	15.8	3.0	5
CS28685	4.8	1.1	5	CS76112	15.9	2.4	8
CS76259	5.4	1.7	5	CS28345	15.9	2.2	9
CS28645	5.6	1.7	5	CS28800	16.0	2.9	5
CS76260	5.6	1.5	5	CS76107	16.0	1.1	7
CS76274	5.7	3.2	3	CS76166	16.0	1.9	5
CS28786	6.0	1.4	5	CS76281	16.0	3.0	5
CS76283	6.0	2.1	3	CS76143	16.2	1.2	5
CS76288	6.0	1.1	5	CS76242	16.2	1.7	5
CS28158	6.2	1.0	5	CS76239	16.3	2.0	4
CS76159	6.2	1.0	5	CS28091	16.4	2.1	5
CS76180	6.2	1.9	5	CS28193	16.6	2.3	5
CS76299	6.2	1.1	5	CS76213	16.6	2.3	5
CS28133	7.0	1.0	5	CS28141	16.8	3.6	4
CS76111	7.0	2.3	4	CS76120	16.8	2.9	5
CS76145	7.0	1.1	5	CS76147	16.8	2.7	5
CS76198	7.0	2.8	5	CS76177	16.8	2.9	5
CS76254	7.0	2.3	10	CS76216	16.8	2.6	5

CS76273	7.0	1.4	5	CS76226	16.8	1.9	5
CS28787	7.2	1.3	5	CS28457	17.2	2.3	5
CS76204	7.2	2.5	5	CS76142	17.2	0.8	5
CS28018	7.4	0.9	5	CS28663	17.3	4.0	4
CS76183	7.4	0.9	5	CS76151	17.3	2.8	4
CS28160	7.5	3.9	4	CS76241	17.5	0.5	2
CS28064	7.6	2.4	5	CS28833	17.6	3.5	5
CS76185	7.6	1.2	5	CS76101	17.6	3.0	5
CS76195	7.6	0.9	5	CS28054	17.7	4.5	7
CS28620	7.8	1.7	5	CS76270	17.8	3.7	5
CS76139	7.8	1.4	5	CS28049	18.0	2.5	5
CS76149	8.2	1.5	9	CS28847	18.0	4.0	4
CS28208	8.4	1.8	5	CS76093	18.0	1.5	3
CS76161	8.4	1.0	5	CS76153	18.0	6.6	5
CS76184	8.4	0.8	5	CS76308	18.0	2.5	4
CS76205	8.7	0.8	6	CS28243	18.2	2.4	5
CS76084	8.7	1.3	7	CS76302	18.2	1.4	5
CS28013	8.8	3.8	4	CS28108	18.3	3.1	4
CS76119	8.8	1.7	5	CS28241	18.4	3.6	5
CS28622	9.0	1.7	5	CS28564	18.4	2.8	5
CS28637	9.0	1.3	5	CS28848	18.4	2.8	5
CS76127	9.0	2.0	5	CS76258	18.4	2.1	7
CS28181	9.2	1.0	5	CS76133	18.6	1.7	5
CS76125	9.2	3.4	5	CS76191	18.6	3.7	5
CS76135	9.2	1.7	5	CS76244	18.6	2.6	5
CS76208	9.2	3.4	5	CS76164	18.7	1.9	19
CS76187	9.3	1.9	4	CS76225	18.8	0.9	5
CS76257	9.3	1.7	4	CS28580	18.9	2.3	9
CS76110	9.4	2.6	5	CS76296	19.2	1.5	5
CS76122	9.4	2.1	5	CS28051	19.3	5.1	4
CS76162	9.4	0.7	5	CS76220	19.4	1.5	5
CS76196	9.4	3.8	5	CS76306	19.4	1.3	5
CS76215	9.4	1.3	5	CS28128	19.5	4.7	4
CS76088	9.6	1.5	5	CS28788	19.7	3.2	3
CS76090	9.6	1.7	5	CS28350	19.7	2.7	7
CS76297	9.6	2.2	5	CS28280	19.8	3.0	4
CS28014	9.8	1.2	5	CS28780	19.8	2.0	5
CS28613	9.8	2.1	5	CS76163	19.8	5.6	5
CS28636	9.8	1.4	5	CS28779	20.0	4.8	5
CS76137	9.8	1.2	5	CS76229	20.0	1.8	4
CS28214	10.0	1.4	5	CS76266	20.0	2.7	5
CS28692	10.0	1.5	5	CS76294	20.0	2.6	3
CS28810	10.0	1.9	5	CS76201	20.2	6.6	5
CS76175	10.0	2.5	3	CS76264	20.2	1.8	5
CS76129	10.2	2.7	5	CS76108	20.3	2.3	8

CS76136	10.2	1.8	5
CS28007	10.3	2.9	4
CS28097	10.3	1.2	3
CS28795	10.4	2.0	5
CS76134	10.4	1.0	5
CS76256	10.4	2.6	5
CS28635	10.6	0.7	5
CS28236	10.8	1.7	5
CS28633	10.8	1.9	5
CS76096	11.0	2.0	7
CS76210	11.0	1.5	5
CS76271	11.0	2.7	5
CS76275	11.0	3.4	5
CS76285	11.0	3.0	5
CS28804	11.2	1.6	10
CS76098	11.2	1.5	5
CS76304	11.3	3.7	4
CS28142	11.6	1.1	5
CS28202	11.6	3.1	5
CS76123	11.6	1.7	5
CS76176	11.6	1.3	5
CS28277	11.8	3.5	5
CS76160	11.8	1.5	5
CS76168	11.8	1.7	5
CS76103	12.0	1.6	4
CS76113	12.2	0.4	455
CS28725	12.2	1.9	5
CS76209	12.2	3.8	5
CS76276	12.2	3.4	5
CS76221	12.3	1.9	4
CS28282	12.4	1.7	5
CS28628	12.4	1.1	5
CS76250	12.4	3.1	5
CS28651	12.5	1.3	4
CS28713	12.6	2.2	5
CS28734	12.6	0.7	5
CS76095	12.6	3.2	5
CS76148	12.6	0.4	5
CS76152	12.8	0.9	4
CS28163	12.8	0.7	5
CS28252	12.8	1.2	5
CS76158	12.8	1.4	5
CS76182	12.8	2.1	5
CS76243	12.8	1.9	5
CS76115	12.9	2.1	8

CS76200	20.4	2.1	8
CS76089	20.4	4.2	5
CS76233	20.6	1.7	7
CS76170	20.6	1.5	5
CS28729	20.8	1.5	5
CS76169	20.8	1.7	5
CS76286	20.8	1.7	5
CS76303	21.0	4.3	5
CS28063	21.3	5.1	4
CS28614	21.3	2.6	12
CS76172	21.3	4.6	6
CS76140	21.4	2.7	5
CS76188	21.4	3.2	5
CS76173	21.5	4.4	6
CS28090	21.6	1.8	5
CS76217	21.8	2.8	4
CS28759	21.8	3.1	5
CS28573	21.9	2.5	10
CS28809	22.0	3.5	5
CS76144	22.0	3.0	8
CS76156	22.0	1.8	10
CS76171	22.0	2.8	5
CS76253	22.0	2.4	5
CS28490	22.1	1.9	7
CS76100	22.2	3.6	6
CS76202	22.2	6.7	5
CS76218	22.2	3.7	5
CS76269	22.2	2.1	5
CS28823	22.4	1.3	5
CS28407	22.5	1.2	4
CS28459	22.6	2.0	10
CS76261	22.6	1.7	5
CS76189	22.8	2.3	5
CS76118	23.0	NA	1
CS76263	23.4	4.2	5
CS76247	23.5	3.0	4
CS76234	23.6	4.4	5
CS76301	23.7	0.9	3
CS76193	23.8	3.5	12
CS76240	24.0	2.0	5
CS76298	24.0	NA	1
CS76105	24.1	4.5	8
CS28812	24.2	4.8	5
CS28758	24.4	2.9	5
CS76086	24.4	6.3	5

CS76138	13.0	1.8	5
CS76272	13.0	1.1	5
CS28135	13.2	3.4	5
CS28165	13.2	1.9	5
CS76087	13.2	1.4	5
CS76228	13.2	1.4	5
CS76248	13.2	1.1	5
CS76223	13.4	3.0	5
CS76128	13.5	1.3	8
CS28640	13.6	4.1	9
CS28626	13.6	1.6	5
CS76094	13.6	3.5	5
CS76121	13.6	3.7	5
CS76126	13.8	1.5	13
CS28274	13.8	0.6	5
CS76099	13.8	4.1	5
CS28201	14.0	1.3	5
CS76106	14.0	4.6	4
CS76132	14.0	2.9	5
CS76277	14.0	1.7	4
CS76097	14.1	4.3	7
CS28650	14.2	1.6	5
CS28724	14.2	3.4	5
CS28268	14.4	1.4	5
CS28343	14.4	3.2	5
CS28583	14.4	3.1	5
CS28631	14.4	1.8	5
CS76267	14.4	1.4	5
CS28217	14.5	2.2	4
CS28017	14.6	3.2	5
CS76109	14.6	2.7	5
CS76291	14.6	2.9	5
CS76083	14.8	2.3	5
CS76245	14.8	2.7	5
CS76279	14.8	2.0	5
CS28595	15.0	3.4	4
CS28739	15.0	1.9	5
CS76146	15.0	1.9	5
CS76199	15.0	1.7	4
CS76230	15.0	4.0	5
CS76104	15.1	2.2	8
CS28527	15.2	1.4	10
CS28610	15.2	3.0	5
CS28822	15.2	4.6	5
CS76141	15.2	1.7	5
CS28279	24.5	5.7	4
CS28808	24.8	3.2	5
CS76292	25.0	0.3	5
CS28513	25.4	1.8	8
CS76295	25.4	2.9	5
CS28420	25.6	3.2	5
CS28336	25.7	4.8	3
CS28344	26.0	6.7	5
CS28720	26.0	1.8	5
CS28814	26.0	5.9	5
CS76214	26.0	2.3	5
CS76222	26.0	3.4	5
CS76255	26.0	7.3	5
CS76232	26.3	1.3	4
CS76280	26.3	3.4	4
CS76293	26.3	3.3	4
CS76284	26.3	5.8	3
CS28760	26.4	5.4	5
CS28743	26.6	3.0	5
CS76238	26.6	3.5	5
CS76212	26.8	3.4	5
CS76287	26.8	5.7	5
CS76092	27.0	5.0	5
CS76207	27.0	NA	1
CS76235	27.0	6.3	5
CS28394	27.2	4.6	5
CS76219	27.2	3.5	5
CS28369	27.9	1.8	9
CS76085	28.0	5.0	5
CS76282	28.0	4.4	5
CS28495	28.4	3.8	5
CS28750	28.4	2.9	5
CS76167	28.6	1.9	5
CS76231	28.6	1.1	5
CS76224	28.8	5.1	5
CS76265	28.8	4.4	5
CS28587	28.9	1.7	10
CS28332	29.7	3.8	3
CS76249	30.2	3.3	5
CS76236	30.6	3.8	5
CS76251	31.4	4.8	5
CS28395	31.6	4.6	5
CS76252	32.0	NA	1
CS28326	33.0	3.1	4
CS76307	33.9	3.6	8

CS76165	15.2	2.4	5	CS76227	35.0	2.5	5
CS76278	15.2	2.8	5	CS76116	35.2	3.5	25
CS76305	15.2	1.8	5	CS76211	35.2	3.6	5
CS76154	15.3	2.2	4	CS28492	35.3	3.8	4
CS76300	15.3	2.3	10	CS28423	36.3	3.8	4
CS76131	15.4	3.6	5	CS28419	38.4	3.3	5
CS76091	15.5	1.4	4	CS28373	39.5	3.1	4
CS76290	15.5	4.1	4	CS76197	39.6	4.3	5
CS28732	15.6	2.9	5	CS28461	39.8	4.3	5
CS76150	15.6	1.8	5	CS28575	40.0	2.5	5
CS76157	15.6	2.5	5	CS28364	40.3	4.1	10
CS76194	15.6	1.3	5	CS22689	41.3	5.1	4
CS76203	15.6	1.5	5	CS28578	42.0	1.0	2
CS28053	15.7	3.8	3	CS28382	42.2	2.8	5
